# Natural mercury exposure of European insectivorous bats may exceed a recognized toxicity threshold

**DOI:** 10.1007/s10646-024-02785-5

**Published:** 2024-07-19

**Authors:** Hana Bandouchova, Kamila Novotna Kruzikova, Jan Zukal, Petr Linhart, Jana Sedlackova, Lucie Veitova, Vendula Kalocsanyiova, Jiri Pikula, Zdenka Svobodova

**Affiliations:** 1 Department of Ecology & Diseases of Zoo Animals, Game, Fish and Bees, University of Veterinary Sciences Brno, Brno, Czech Republic; 2 Department of Animal Protection and Welfare & Veterinary Public Health, University of Veterinary Sciences Brno, Brno, Czech Republic; 3 Institute of Vertebrate Biology, Czech Academy of Sciences, Brno, Czech Republic

**Keywords:** Carabidae, Central Europe bats, Chiroptera, heavy metals, *Myotis myotis*, tissue analysis

## Abstract

Heavy metals are an important group of toxic substances harmful for many organisms. Of these, mercury is one of the most monitored in the environment. Several matrices are used for the monitoring of environmental load, including a range of organisms; bats, however, have only been examined rarely. Insectivorous bats are apex predators threatened by several human interventions in their natural environment, including heavy metal pollution. The aim of this study was to analyze the content of total mercury in the fur, flight membrane, and pectoral muscle of greater mouse-eared bats (*Myotis myotis*). Total mercury concentrations were also measured in carabid beetles from the catch locality Zastávka u Brna. Samples were obtained from 43 bat carcasses at two different localities in the Czech Republic (Zastávka u Brna, Malá Morávka). Total mercury content varied between 1.76–72.20 µg/g in fur, 0.04–0.14 µg/g in skin, and 0.05–0.20 µg/g in muscle. Total mercury values in the fur of some individuals from Malá Morávka exceeded the recognized toxicity limit. Furthermore, there was a significant difference (*p* < 0.001) in content of total mercury in fur between localities, and there was a clear effect of age on concentrations in fur, skin, and muscle, the concentrations being significantly correlated (fur and skin r_s_ = 0.783; fur and muscle r_s_ = 0.716; skin and muscle r_s_ = 0.884). These findings confirm the usefulness of fur samples from living bats for biomonitoring mercury burden in the environment.

## Introduction

Heavy metals are an important group of toxic substances in the environment as many can bioaccumulate in the tissues of wild organisms (Ahmad et al. 2021, Morais et al. 2012), the metals continuously accumulating in their bodies when they consume contaminated food or water (Pikula et al. 2013; Wren 1986). Heavy metals are known to interfere with reproduction in wildlife, causing damage to reproductive organs and reducing fertility (Abdelsalam et al. 2021) and can also affect immune responses to infection (Becker et al. 2021). However, the impacts of heavy metals on wildlife do not necessarily have to be in the form of acute toxicity. In several areas, wild animals have been exposed to relatively low, long-term doses, and this chronic exposure can still result in physical damage and a decrease in overall fitness, and thus can threaten the survival of wildlife populations (Powolny et al. 2023).

Exposure to mercury has a significant effect on human and animal health, including wildlife (Wolfe et al. 1998). Contamination of some locations has been directly related to human activity, and such sites can represent a serious health problem. One important source of mercury is deposition associated with coal mining, combustion, and industrial production (Driscoll et al. 2013; Selin et al. 2009; Boeing 2000). Biomagnification of mercury in food chains is a serious problem, with some apex predators being exposed to very high doses (Zhang et al. 2022; Lavoie et al. 2013; Knopf and König, 2010) The effects of mercury will depend on the species involved, the chemical form of mercury (e.g., elemental, inorganic, or methylmercury), dose, duration of exposure, and the age and health of the exposed individual (Rice et al. 2014). Mercury in its methylmercury form is particularly harmful to animals as it can damage the nervous system, disrupt reproductive processes, and cause developmental abnormalities (Massányi et al. 2020; Cariccio et al. 2019; Mobarak 2008).

Bats are an interesting group of highly specialized mammals. Bats are considered important bioindicators due to the great variability in types of food taken, meaning they can occupy different trophic levels in an ecosystem (Yates et al. 2014; Jones et al. 2009). Bats feeding on insects contaminated with heavy metals may bioaccumulate these metals in their tissues in the same way as insectivorous birds or shrews (Jackson et al. 2015; Ageeva et al. 2023). Analyzing the concentration of heavy metals in bat tissues, as well as their fur and droppings, could provide insights into the metal contamination of their foraging areas (Timofieieva et al. 2021; Pikula et al. 2010); however, the migration distances of individual species should be considered in such cases. Furthermore, bats often have longer lifespans than other small mammals, making them potentially useful for tracking changes in heavy metal exposure over time, i.e., their dietary habits could provide insights into spatial and temporal variations in heavy metal pollution (Zukal et al. 2015). Interestingly, while previous studies have shown increasing mercury concentrations in the tissues of bats living near a mercury source, increased concentrations have also been recorded in bats from environments with no known mercury source (Korstian et al. 2018; Ferrante et al. 2018; Little et al. 2015).

The digestive system, along with inhalation, represents the main route of entry into the body for mercury, with food as the primary source. The greater mouse-eared bat (*Myotis myotis*), a large Palearctic bat species, is a typical “surface gleaner”, hunting mostly on the ground or collecting prey from plant surfaces (Arlettaz et al. 1997; Pereira et al. 2002). The main dietary items taken tend to be carabid beetles, particularly those of the genera *Carabus* and *Pterostichus* (Jaskuła and Hejduk 2005; Graclik and Wasielewski 2012). The Carabidae are a diverse group of epigeic and mostly predatory beetles, that have been shown to bioaccumulate mercury in their bodies (Šerić Jelaska et al. 2014). The aim of this study, therefore, was to (1) evaluate concentrations of total mercury in the fur, flight membrane, and pectoral muscle of *M. myotis* from the Czech Republic (Central Europe), (2) to determine the effect of age and gender on the content of total mercury in the monitored tissues, and (3) to compare concentrations of total mercury in the fur of *M. myotis* from two different localities. At the same time, we also measured the concentration of total mercury in carabid beetles collected at one of the localities. We hypothesized that there would be (1) higher total mercury concentrations in older individuals due to bioaccumulation, (2) higher concentrations in females due to higher food intake during pregnancy and lactation, (3) a correlation between content of total mercury in fur and other matrices, and (4) species-specific differences in concentrations of total mercury in different carabid beetles.

## Material and Methods

### Study sites

Our main study site was situated at Zastávka u Brna (49.1875297N, 16.3589492E), a town about 15 km west of Brno, South Moravia, Czech Republic. Coal was discovered in this area in the second half of the 18th century and mining began in 1788 and ended in 1967. Our second site was located at the Šimon and Juda iron-stone mine near the village of Malá Morávka site, specifically the Šimon and Juda iron-stone mine (50.0512206N, 17.2980758E) in the Jeseníky region (North Moravia, Czech Republic), adjacent to the Ostrava-Karvina basin industrial and coal mining area and the neighboring coal mining region in Poland. The caves and mines in this region represent important *M. myotis* hibernation sites, while summer colonies of these bats roost in the attics of buildings.

### Samples

A total of 43*M. myotis* carcasses were collected from the two localities, 32 from attics in Zastávka u Brna at the beginning of August and 11 from an old mine near Malá Morávka at the end of hibernation. Sampling took place in cooperation with the Institute of Vertebrate Biology of the Czech Academy of Sciences (Permits 181108/2016/KUSK, JMK 13759/2016, KUJCK 161737/2016/OZZL, 3640/ZPZ/2016/ZD-893, KUZL 68700/2016, according to Decree No. 395/1992 Coll., Annex III). Following collection, all bats were sexed, and samples for further analysis were collected during necropsy.

Total mercury content was assessed on fur, in-flight membrane (herein skin), and pectoral muscle (herein muscle) from the 32*M. myotis* from Zastávka u Brna, and in fur samples only from the 11 Malá Morávka bats. Fur was clipped from the dorsal part of the body and the samples were stored in paper bags, while skin and muscle samples were stored in zipped polyethylene bags. All samples were then frozen at −18 °C until further analysis. Bats from Zastávka u Brna were also classified into three age categories based on the degree of canine teeth attrition, with group 1 (*n* = 10) corresponding with group 1C in Schick et al. (2003); group 2 (*n* = 12) corresponding with groups 2C and 3C, and group 3 (*n* = 10) corresponding with groups 4C and 5C.

To assess concentrations of total mercury in *M. myotis* prey, three potential foraging sites (49.1875572N, 16.3738756E; 49.1469681N, 16.3475308E; 49.1874486N, 16.3738164E) was chosen close to the bat colony in Zastávka u Brna and carabid beetles caught using pitfall traps filled with saturated saline solution. The carabid beetles caught were determined to species (Hůrka 1996) and then stored frozen at −18 °C until further analysis.

### Mercury analysis

Total mercury content was measured (µg/g of fresh tissue (ww)) in the collected tissue samples (muscle and flight membrane) using an AMA 254 trace mercury analyzer (Altec Ltd., Prague, Czech Republic) without prior sample preparation, while fur was washed (1x in acetone, 3× in water, and 1x in acetone) to remove surface contamination before measurement. The samples were first thawed, then approximately 50 mg of muscle or 20 mg of fur or skin were weighed on Precisa 125 A analytical scales (Dialab Praha, Czech Republic), placed into combustion boats, and inserted into the AMA 254 analyzer with no further sample preparation. The samples were then dried at 120 °C for 60 s and thermally decomposed at 550 °C for 150 s under oxygen flow. Prior to analysis, the carabid beetles were rinsed with water, then acetone, and finally rinsed again with water. The cleaned beetles were then dried at room temperature for five days and then homogenized in a mortar. In the case of small specimens, mixed samples comprising the same species from the same locality were prepared. Subsequently, samples of known weight were assessed using the AMA 254 analyzer, where the samples were dried at 120 °C for 10 s and thermally decomposed at 550 °C for 100 s under oxygen flow. All results are presented as dry weight.

The AMA 254 trace mercury analyzer is a single-purpose atomic absorption spectrometer that uses the principle of vapor generation of metallic mercury through thermal decomposition of the sample in a combustion tube, with subsequent capture and concentration on a gold amalgamator, thermal re-expulsion, and detection. The AMA 254 analyzer works in two ranges, an automatic switch-over for lower and higher mercury concentrations (0.05–40 and 40–500 ng Hg). For calibration, we used an AA standard comprising 1000 µg/mL mercury in 5% nitric acid (Agilent Technologies, USA). The accuracy of measurement is <1.5% the detection limit is 0.18 µg/kg the limit of quantification (LOQ) is 0.59 µg/kg (limit of detection (LOD) is three times the standard deviation (SD) of a blank and the LOQ ten times the blank SD). Each sample was measured at least twice, and if the relative standard deviation (RSD) of the measurement of duplicate samples was higher than 10%, other parts of the sample were measured until the RSD was <10%, as stated in the manufacturer’s instructions. For validation, we used the standard reference material (SRM) NIST 2976 (National Institute of Standards and Technology, Gaithersburg). Recovery was determined by SRM 10 times measurement at 94.8%.

### Statistical analysis

Correlation analysis was undertaken using the STATISTICA 12 software package (StatSoft, Inc., USA). To assess the significance of factors influencing the concentration of total mercury in fur, muscle, and skin of *M. myotis* bats from Zastávka u Brna, in carabid beetles from foraging sites close to Zastávka u Brna and in the fur of *M. myotis* bats from Zastávka u Brna and from Malá Morávka, we used general linear models (GLM) or generalized linear models (GLZ), performed in R version 4.2.1 (R Core Team, 2022), For total mercury in fur, muscle and skin of bats from Zastávka u Brna, we used the GLM formula total_mercury_in_matrix ~ age*sex as the maximal model; for total mercury in carabid beetles, a maximal GLM model was used based on the formula total_mercury ~ species_of_carabidae*sampling_locality (singletons excluded). Based on the results, non-significant factors were removed one by one, and the minimal model was used for analysis. For total mercury in fur, a GLM model was used based on the formula total_mercury ~ locality. In cases of heteroscedasticity, a GLZ was used with settings Gamma(link = log) or a GLM with logarithmic transformation of data. For individuals from Zastávka u Brna, data normality was initially tested using the Shapiro-Wilk test, while the Spearman rank correlation coefficient was used to evaluate correlations between total mercury values in the different matrices.

## Results

Total mercury concentrations in fur ranged from 1.76 to 72.20 µg/g (median 5.19 µg/g) in bats from both localities, with values ranging from 1.76 to 13.82 µg/g (median 3.66 µg/g) for Zastávka u Brna and 5.19 to 72.20 µg/g (median 30.97 µg/g) for Malá Morávka (Table 1). For skin and muscle samples from Zastávka u Brna, concentrations in skin ranged from 0.04 to 0.14 µg/g (median 0.07 µg/g) and 0.05 to 0.20 µg/g (median 0.10 µg/g) in muscle (Table 1). For carabidae, concentrations ranged from 0.01 in *Platynus assimilis* to 0.18 µg/g in *Pterostichus melanarius*, with a median of 0.03 µg/g (Table 2).

**Table 1:** Total mercury concentrations (min-max and median in µg/g) in *Myotis myotis* fur, skin, and muscle samples from the two study sites.

		total mercury		
Sites	*N*	Fur	Skin	Muscle
Zastávka u Brna	32	1.76–13.82	0.04–0.14	0.05–0.20
		3.66	0.07	0.1
Malá Morávka	11	5.19–72.2	NA	NA
		30.97		

NA = not applicable

**Table 2:** Total mercury concentrations (min-max and median in µg/g) in carabid beetles from Zastávka u Brna (Note: species with just one occurrence were removed from further analysis)

		whole body total mercury
species	*N*	median
*Carabus ullrichii*	9	0.03–0.1
		0.06
*Carabus violaceus*	4	0.08–0.12
		0.09
*Carabus scheidlerii*	2	0.02–0.03
		0.03
*Brachinus crepitans*	5	0.04–0.09
		0.06
*Harpalus latus*	11	0.01–0.04
		0.02
*Harpalus rufipes*	5	0.02–0.03
		0.02
*Platynus assimilis*	11	0.01–0.06
		0.02
*Cicindela germanica*	1	0.03–0.03
		0.03
*Pterostichus melanarius*	1	1.18–1.18
		1.18

**Fig. 1:**
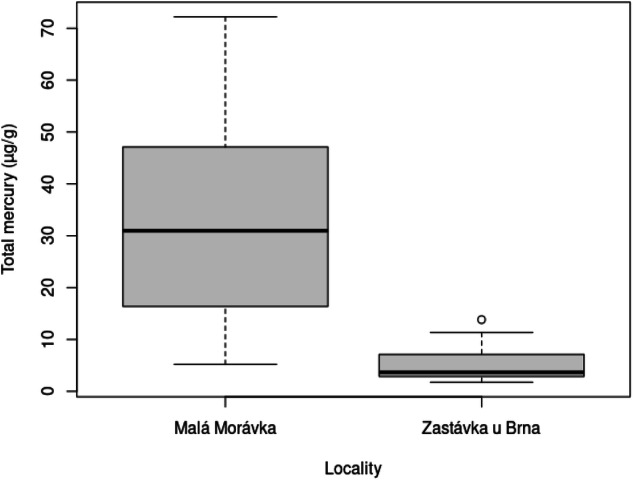
Box plot of total mercury concentrations in the fur of *Myotis myotis* from the two study sites (GLZ (Gamma(link = log), *p* < 0.001). Box plots show the 1st and 3rd quartiles and the median value; the whiskers show 1.5 times interquartile range, and circles indicate outliers

**Fig. 2:**
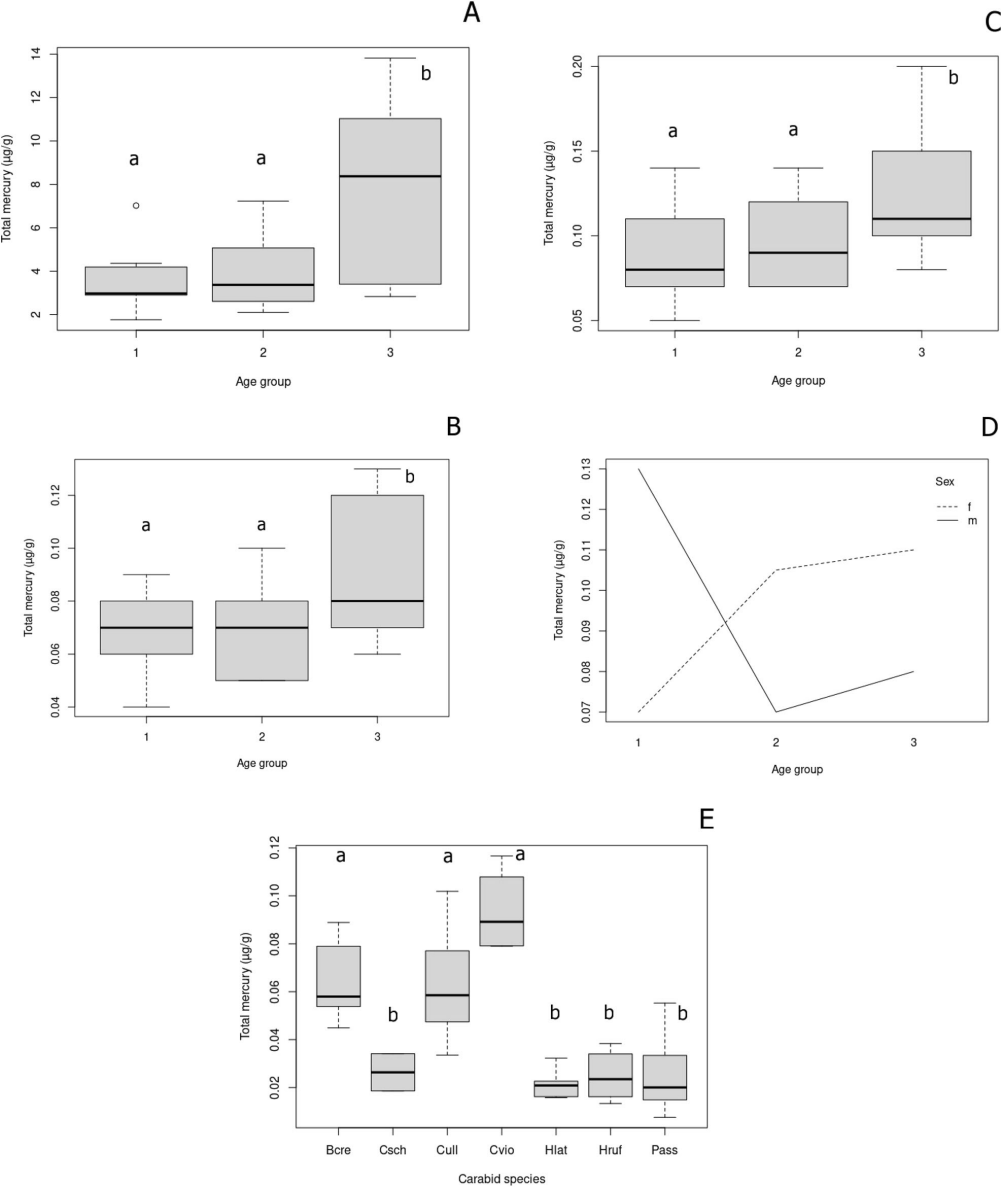
Total mercury concentrations in fur (**A**; *p* < 0.001), skin (**B**; *p* < 0.01), and muscle (**C**; *p* < 0.05) samples; interaction of sex and age in muscle concentrations (**D**), and concentrations in carabid beetles from Zastávka u Brna (**E**; *p* < 0.001). Box plots show the 1st and 3rd quartiles and the median value; the whiskers show a 1.5 times interquartile range, and circles indicate outliers. Age group: group 1 ≡ group 1C in Schick et al. (2003), group 2 ≡ groups 2C and 3C, group 3 ≡ groups 4C and 5C; Carabid species: Bcre = *Brachinus crepitans*, Csch = *Carabus scheidlerii*, Cull = *Carabus ulrichii*, Cvio = *Carabus violaceus*, Hlat = *Harpalus latus*, Hruf = *Harpalus rufipes*, Pass = *Platynus assimilis*

The final minimal model for concentration of total mercury in fur was a GLZ with Gamma distribution and a logarithmic link function that included locality as a variable (*p* < 0.001), the model explaining 68.38% of data variability (Fig. 1). Owing to heteroscedasticity in the data, a GLZ with Gamma distribution and logarithmic link function was also the best fitting model for analyzing factors influencing concentrations in fur, muscle and skin in bats from Zastávka u Brna. The minimal model for fur and skin, which included age categories 1–3 as a factor, explained 42.74% (*p* < 0.001) and 22.23% (*p* < 0.05) of data variability, respectively, with a highly significant difference found between age groups 1 and 3 (*p* < 0.001) and no difference between groups 1 and 2 (Fig. 2a, b). The minimal model for concentrations in muscle, which included age categories 1–3 (Fig. 2c; *p* < 0.05) and the age: sex interaction (Fig. 2d, *p* < 0.05) as factors, explained 43.05% of data variability, with a significant difference between age groups 1 and 3 but no difference between groups 1 and 2. In age group 1, concentrations in muscle were significantly higher in males, while in groups 2 and 3 concentrations were significantly higher in females (Fig. 2d). The minimal model for concentrations in carabid beetles was a GLZ model with Gamma distribution and logarithmic link function with species included as a factor (Fig. 2e, *p* < 0.001). As part of the comparison, species at a higher trophic level, i.e., those feeding on earthworms or larvae of other ground beetles and species feeding on plant food and small invertebrates, were compared using individually set contrasts, with a highly significant difference found between the two groups (*p* < 0.001).

**Fig. 3:**
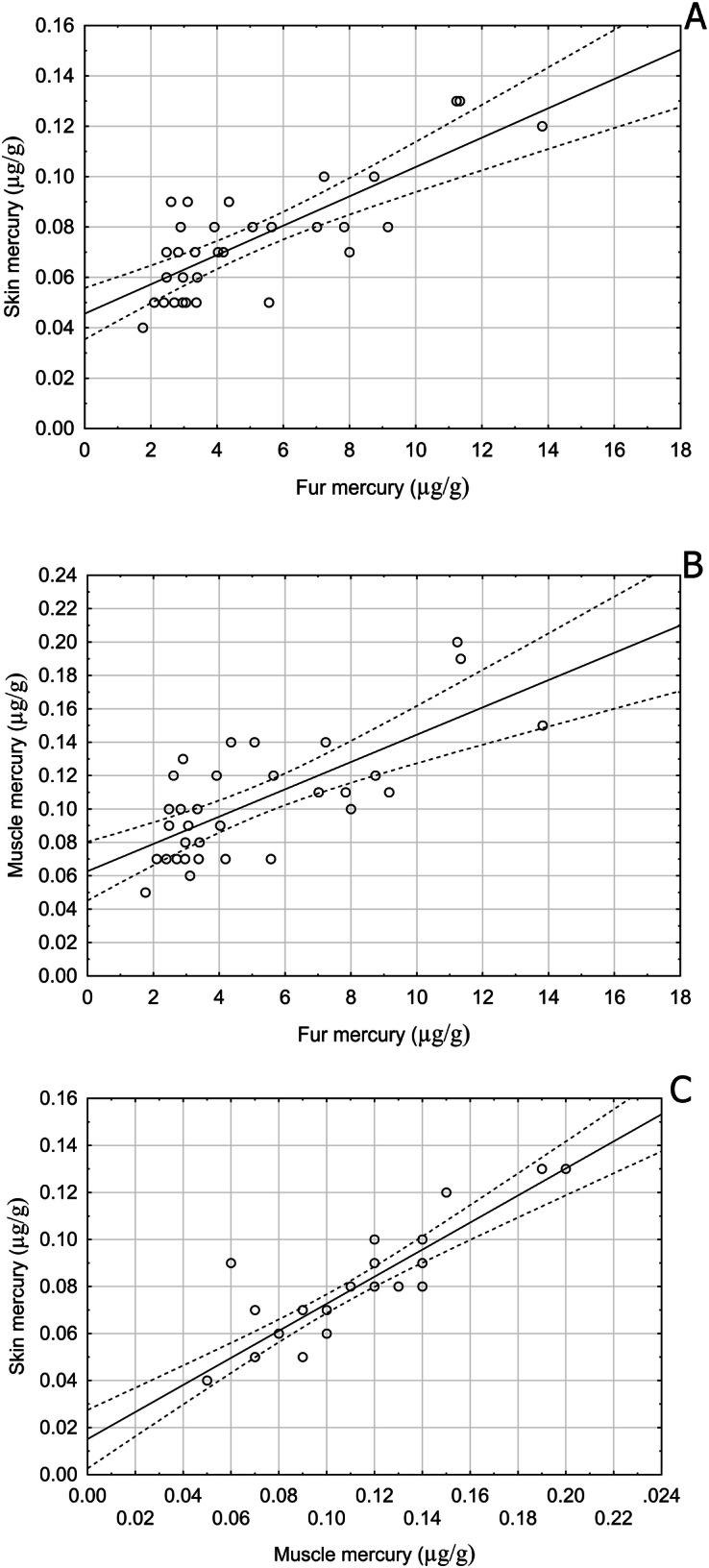
Correlation between total mercury concentration in (**A**) skin and fur (Spearman rank correlation r_s_ = 0.783, *p* < 0.001), (**B**) muscle and fur (r_s_ = 0.716, *p* < 0.001), and (**C**) skin and muscle (r_s_ = 0.884, p < 0.001) in *Myotis myotis* from Zastávka u Brna. Dashed lines = 95% confidence intervals

As the Shapiro-Wilk test revealed non-normal distribution of data, the non-parametric Spearman rank correlation was used for further analysis, revealing a highly significant difference between the matrices from Zastávka u Brna (Kruskal-Wallis, H = 156,533, *p* < 0.001), with total mercury decreasing as follows: fur > muscle > skin. Furthermore, a significant positive correlation was observed between concentrations in individual matrices, with fur and muscle at r_s_ = 0.716, fur and skin at r_s_ = 0.783, and muscle and skin at r_s_ = 0.884 (Fig. 3a–c; all *p* < 0.05).

## Discussion

Bats, with their long life-span and strong fidelity to specific localities, represent a suitable group of animals for biomonitoring environmental pollution. However, all European species are subject to strict protection, which is one reason why there has only been a limited amount of work dealing with pollutant concentrations in the tissues of these insectivorous mammals (Timofieieva et al., 2021; Ferrante et al., 2018; Lisón et al., 2017; Zukal et al., 2015; Pikula et al., 2010). Samples taken from dead individuals, however, present a unique opportunity to assess bat exposure to pollutants. It should be noted, however, that taking samples from dead individuals entails the risk that the pollutants contributed to the death of the individual and that their concentration may not correspond to normal concentrations in other individuals of the given species in the locality investigated (Mierle et al., 2000).

Both previous studies and our own confirm an affinity between mercury and fur (e.g. Mina et al., 2019), thereby offering the possibility of monitoring heavy metals based on non-lethal live sampling, which is more likely to reflect exposure in a given location. Furthermore, if values in fur correlate with concentrations in other tissues, the results from fur analysis could then be used to estimate concentrations in internal organs. As absorbed mercury is distributed from the blood to other tissues, where detoxification or elimination takes place, concentrations in blood and muscle will reflect acute exposure, while concentrations in fur will correspond to exposure during hair growth (Hernout et al., 2016). As an example, concentrations of total mercury in blood were strongly correlated with levels in muscle in northern elephant seals (*Mirounga angustirostris*); however, the correlation was weaker for fur and blood and fur and muscle (Peterson et al., 2016).

In our *M. myotis* samples from Zastávka u Brna, we observed significant correlations between all matrices examined, i.e. fur, skin, and muscle. As mercury concentrations in fur and skin or muscle were very strongly correlated, we were subsequently able to use fur analysis to assess levels of exposure and risk of negative effects, with the proviso that, since bats move between their hibernation sites and summer colonies during the year, interpretation must consider when the sample was collected, e.g. before or after the species’ molting period (Fraser et al., 2013). In addition, we observed a significant intraspecific difference in mercury concentrations in *M. myotis* fur when comparing our two study localities, with a max. concentration of 13.82 µg/g (median 3.66 µg/g) in the area where coal was no longer mined (Zastávka u Brna) and max. 72.2 µg/g (median 30.97 µg/g) in the area with ongoing coal mining and industrial enterprises (Malá Morávka). The highest measured concentration (72.2 µg/g) in our study was surprisingly high, even when compared with other studies examining total mercury in bat fur (Yates et al., 2014; Åkerblom and de Jong, 2017; Lisón et al., 2017; Kieffer et al., 2023).

The Malá Morávka site is situated within the Jeseníky Mountains protected area and represents an important hibernation site for the species. As concentrations of total mercury in fur are most likely to correspond with the active part of the animal’s annual life-cycle, i.e., the period of fur growth (Hernout et al., 2016), and as *M. myotis* undertakes relatively short flights (usually up to 150 km, only exceptionally 200 to 300 km; Gaisler et al., 2003; Wojtaszyn et al., 2014), we assume that the *M. myotis* hibernating around the Jeseníky Mountains arrive from either the central Moravian region, the Ostrava-Karvina coal mining and industrial region or a similar region in nearby Poland (Gaisler et al., 2003). Despite this, we were unable to definitively relate the concentrations of total mercury detected in the fur of bats from Malá Morávka to exposure at a specific location. Of particular interest, some animals at both Zastávka u Brna and Malá Morávka displayed concentrations in fur higher than the limit of 10 µg/g considered the toxicity threshold for small mammals (Burton et al., 1977; Nam et al., 2012), thereby indicating high mercury exposure.

According to Rand et al. (2020), the amount of mercury in blood corresponds with an animal’s acute exposure to this heavy metal. When an animal ingests mercury, the body attempts to remove it using a variety of mechanisms, including excretion in feces or urine (Clarkson et al., 2007). On the other hand, once in the body, mercury may be retained by elements such as selenium (Romero et al., 2016; Parizek and Ostadalova, 1967) or cysteine-rich proteins such as metallothioneins, that can bind to elements such as heavy metals (Pikula et al., 2010; Morcillo and Santamaria, 1993). As discussed above, a further important route of mercury elimination is deposition in the fur (Jota Baptista et al., 2022). The high fur mercury concentrations in our *M. myotis* group 3 individuals (i.e., older individuals) from Zastávka u Brna suggest long-term bioaccumulation and/or a reduced detoxification capacity, perhaps through reduced metallothionein production. Interestingly, higher mercury concentrations were also recorded in the fur of adult (compared to juvenile) arctic foxes (*Vulpes lagopus*; Bocharova et al., 2013), and in the liver, kidney, and brain of adult Franciscana dolphins (*Pontoporia blainvillei*; Romero et al., 2016). Grottoli et al. (2023), on the other hand, found decreasing concentrations of mercury in the fur of repeatedly captured little brown bats *Myotis lucifugus* over the course of six years. In our case, a higher concentration was found in group 3 compared to the other two age groups. Group 3 includes older individuals. In a study by Grottoli et al. (2023), this age group was merged with adult bats which in our study are included in group 2, and their fur mercury concentration does not differ significantly from juvenile animals in group 1.

Total mercury in skin samples from Zastávka u Brna displayed a similar trend to that in fur, presumably due to the close connection between skin tissue and fur (a skin derivative). Analysis of the effect of age and gender on mercury concentration in muscle tissue, however, showed a distinct effect of age, along with a weaker influence of gender. When comparing age groups 1 (juveniles) with groups 2 and 3 (adults), there was an opposing trend visible, with a higher concentration in males than females in group 1, and the reverse in groups 2 and 3. Nielsen and Andersen (1991) also described a higher retention of mercury in the muscles of adult female mice compared with males after a single oral dose of methyl mercuric chloride (1 µmol/kg of body weight). Experimental data in the same study (Nielsen and Andersen, 1991) indicated that adult male mice were able to eliminate mercury from the body more rapidly than females, which is consistent with our own results in adult bats. In the case of juveniles, however, the situation was the opposite, suggesting that the ability to eliminate mercury more effectively develops gradually, as described by Nielsen and Andersen (1996) for mice.

Unlike food intake or air inhalation, the process of mercury excretion is strongly influenced by the detoxification capacity of the animal’s body, with poor excretion leading to an increase in the concentration in blood following exposure. This poses a risk of mercury moving into tissues of the central nervous system (CNS), where it will have a toxic effect. Consequently, the body moves absorbed mercury in the blood to organs that ensure its detoxification and subsequent removal from the body, such as the liver and kidneys. Owing to the limited capacity of these organs, however, there are other tissues in the body that can accumulate mercury and thereby help protect sensitive organs such as the brain. Rand et al. (2020), for example, recorded a 1.7- to 3-times greater concentration of total mercury in muscles compared to blood and brain tissue in mice, which supports the assumption of Thomas et al. (1986) that skeletal muscle serves as a storage organ that absorbs mercury from the blood. Furthermore, the authors detected motor disorders in male mice born to females exposed to high concentrations of methyl mercury chloride, though it was not clear whether this was a result of the action of mercury in the muscles or a toxic effect in the CNS (Rand et al., 2020). In our own study, comparable concentrations of total mercury were found in both muscle and skin, suggesting that the skin may play a similar protective role as muscle in removing mercury from the blood as well as serving as an excretory organ.

The molting period is a significant factor influencing the interpretation of mercury concentrations in fur. In *M. myotis*, new fur growth occurs in males from June to July, and in females and juveniles from late July or early August to late September (Mazak, 1965). Juvenile and subadult animals represent a specific group that may not undergo complete fur replacement during their first year, and even yearlings may still have fur that grew during the period when they were fed with mother’s milk (Fraser et al., 2013). Thus, our results for subadult individuals (group 1 from Zastávka u Brna) may reflect exposure during prenatal development and the milk-feeding period.

In bats at a mercury-contaminated site in Virginia, USA, little brown bats *M. lucifugus* had a total mercury concentration of 274 µg/g in their fur, which is four times the maximum value measured in the present study. A significant level of mercury contamination was later confirmed at this location, which had been discharged into the local river from a manufacturing plant over a long period (Nam et al., 2012). In comparison, significantly lower concentrations were found in the fur of 10 different bat species from the north-eastern part of the USA, the maximum value measured being 3.76 µg/g in *M. lucifugus* (Yates et al., 2014). Korstian et al. (2018) reported mercury concentrations ranging between 1.08–10.52 µg/g in bat fur of different species in the USA, while the highest concentration of total mercury detected in fur in Europe is 14.0 µg/g in whiskered bats *Myotis mystacinus*, 10.4 µg/g in Brandt’s bats *Myotis brandtii* (Kieffer et al., 2023), 2.27 µg/g in a gestating adult common bent-wing bat *Miniopterus schreibersii* female (Lisón et al., 2017) and 2.31 µg/g (Åkerblom and de Jong 2017) in a Daubenton’s bat *Myotis daubentonii*, a water-bound species that preys on aquatic insects and, exceptionally, on small fish (Siemers et al., 2001). In a study conducted in an area with active gold mining in Peru, the highest recorded concentration of total mercury in fur was 8.67 µg/g in the insectivorous/omnivorous lesser spear-nosed bats *Phyllostomus elongatus* (Moreno-Brush et al., 2018).

Analysis of fur samples from 22 bat species from Belize, South America, confirmed the importance of diet as a source of mercury, with the highest concentrations (145.27 µg/g) found in the fur of a piscivorous species, the greater bulldog bat *Noctilio leporinus* (Becker et al., 2018). Levels in this species were one to three orders of magnitude higher than those recorded in the other species studied, with insectivorous species having higher concentrations than carnivorous species, then sanguinivorous species and, finally, frugivorous species, which had the lowest concentrations of total mercury in the fur of any species studied (Becker et al., 2018). Clearly, diet plays an important role in how much mercury enters the body, with a significant difference in the potential rate of mercury bioaccumulation at different trophic levels. Even within insectivorous species, however, small differences in mercury content may be expected in their food since different groups of insects also belong to different trophic levels.

*Myotis myotis* bats are known for their specific hunting style, with a strong specialization for ground-living beetles. The most abundant group in the diet of this bat species are Coleoptera (Graclik and Wasielewski, 2012), and especially Carabidae (ground beetles), which themselves feed on insect prey and thus potentially bioaccumulate heavy metals, including mercury, in their bodies (Šerić Jelaska et al., 2014). As part of the present study, 49 carabid beetles were captured at locations in the immediate vicinity of Zastávka u Brno. Concentrations of total mercury in beetles differed significantly by species, ranging from 0.0076 µg/g in *Platynus assimilis* to 0.1754 µg/g in rain beetles *Pterostychus melanarius*. The highest concentrations were found in the species’ *P. melanarius*, violet ground beetles *Carabus violaceus*, and *Carabus ulrichii*, all favored prey species of *M. myotis* (Arlettaz et al., 1997; Graclik and Wasielewski 2012), which feed largely on earthworms and are known to accumulate mercury in their tissues (Fawki et al., 2003; Šerić Jelaska et al., 2014). In addition, high mercury concentrations were also detected in *Brachinus crepitans*, which, while it does not feed on earthworms, has larvae that parasitize the larvae of other ground beetles (Saska and Honek 2004). Conversely, low mercury concentrations were recorded in the species *Harpalus rufusipes*, *H. latus*, and *Carabus scheidlerii*, which feed on plant seeds (Talarico et al., 2016; Hazarika and Kalita, 2018) and in *P. assimilis* and *Cicindela germanica*, which feed on small insects (Ameixa and Kindlmann, 2008; Else, 1993). The relatively high mercury concentrations in the preferred prey species of *M. myotis* indicate a potentially high level of mercury exposure in this bat species through ingested food.

In the case of chronic exposure, resulting effects will depend on the capacity of the bat’s adaptation mechanisms for mercury detoxification and excretion, with levels exceeding this capacity leading to disruption of the internal environment and the potential death of the affected individual (Nam et al., 2012). As a result, long-term environmental burdens could also have effects at the population level. As bats provide a range of important ecosystem services, such as regulation of invertebrate populations, including agricultural crop pests (Ramírez-Fráncel et al., 2022), any decline in sensitive species could have an impact on biological diversity and disrupt food webs, nutrient cycling, and, in the long-term, functioning of ecosystems (Tovar-Sánchez et al., 2018).

## Conclusion

Our study found significant differences in the content of total mercury in different body matrices, with mercury content decreasing from fur to muscle to skin. Mercury levels were higher in older individuals and in bats from one of the study localities (Malá Morávka); however, a significant interaction between sex and age was only recorded in muscle. Some individuals from both study localities (Malá Morávka and Zastávka u Brna) had total mercury values in the fur exceeding the toxicity limit of 10 µg/g. We also found a strong correlation between concentrations of total mercury in fur, muscle, and skin, suggesting the possibility of using fur for biomonitoring mercury burden in the environment, whether obtained from dead individuals or sampled non-invasively from living bats. Such non-lethal monitoring methods will allow assessment of the current status of mercury pollution and any trends, though it will be necessary to consider the suitability of a given species based on its prey preferences, the distance it migrates, and the species’ molting period. Despite increased monitoring and control of heavy metal pollution in the environment, mercury exposure can still reach levels that threaten insectivorous bats, and, as such, long-term pressure will be crucial in decreasing mercury pollution.
